# It’s all in the timing: Acceptability of a financial incentive intervention for linkage to HIV care in the HPTN 065 (TLC-Plus) study

**DOI:** 10.1371/journal.pone.0191638

**Published:** 2018-02-02

**Authors:** Victoria Shelus, Jamilah Taylor, Elizabeth Greene, Jill Stanton, Allison Pack, Elizabeth E. Tolley, Bernard M. Branson, Wafaa M. El-Sadr, June Pollydore, Theresa Gamble

**Affiliations:** 1 Science Facilitation, HPTN Leadership and Operations Center, FHI 360, Durham, NC, United States of America; 2 Behavioral, Epidemiological and Clinical Sciences, FHI 360, Durham, NC, United States of America; 3 Scientific Affairs, LLC, Atlanta, GA, United States of America; 4 ICAP at Columbia University, Mailman School of Public Health, New York, NY, United States of America; 5 The Women’s Collective, Washington, D.C., United States of America; Brown University, UNITED STATES

## Abstract

The HPTN 065 (TLC-Plus) study tested the feasibility and effectiveness of using financial incentives (FIs) to increase linkage to care (L2C) among individuals with newly diagnosed HIV and those out of care in the Bronx, NY and Washington, DC. Qualitative data collection with a subset of participating patients and staff focused on experiences with and attitudes about the FI intervention. Semi-structured interviews were conducted with 15 patients and 14 site investigators. Four focus group discussions were conducted with a total of 15 staff members. The use of FIs for L2C was generally viewed favorably. Patients were grateful and benefited financially, but sites had some challenges implementing the program. Challenges included the timing and sensitive introduction of the intervention immediately after an HIV diagnosis, negative attitudes towards paying people for health behaviors, and the existence and strength of existing linkage programs. Future programs should consider optimal timing and presentation of FIs.

## Introduction

Linkage to HIV care (L2C) for an HIV-positive person is a pivotal step in the HIV care continuum, and is critical for garnering benefits of care and treatment interventions, as well as for reducing new HIV infections [1]. The benefits of L2C are significant at every stage of HIV disease, but are magnified if this occurs during the early stages. Immediate and effective linkage ensures early access to diverse interventions that prevent HIV-related complications, and disease progression, as well as access to prevention interventions.

Unfortunately, in the United States (US), only 84% of persons who test positive for HIV complete an initial appointment for HIV medical care, and only slightly over half of those with diagnosed HIV are engaged in care [2]. With approximately 1.2 million HIV-positive persons living in the US [2] and about 40,000 diagnoses annually [3], the number of HIV-positive persons who are not in care is substantial. Factors at both individual and system levels; such as mental illness, substance use, HIV stigma and denial, passive referrals post-diagnosis, and insufficient psychosocial support services contribute to this lower proportion of individuals linked to or retained in care, and certain demographic groups, particularly younger persons, women, and racial and ethnic minorities, are associated with poor L2C rates [4, 5]. Given the importance of L2C, it is critical to find methods that will successfully link patients with newly diagnosed infection to care and shorten the time interval between diagnosis and L2C.

### HPTN 065 study and qualitative substudy

The HPTN 065 study evaluated the feasibility of an enhanced community-level **t**est, **l**ink to **c**are, **plus** treat (TLC-Plus) approach for HIV prevention in the US in the Bronx, NY and Washington, DC. This multi-component study was based on the constructs of the mathematical “test and treat” model [6], which hypothesizes that universal voluntary HIV testing, effective L2C, and immediate initiation of ART with high rates of retention in care and adherence to treatment, could dramatically reduce HIV incidence in high-prevalence populations, such as the Bronx and DC. One component of HPTN 065 tested the feasibility and effectiveness of using financial incentives (FIs) to increase L2C among patients with newly diagnosed infection and those who had been out of care for at least one year.

While several studies have explored the use of FIs to encourage HIV testing [7–12], antiretroviral therapy (ART) pill taking [13–16], and viral suppression [17–20], only one study has examined the use of FIs for L2C, and it was specifically among drug users in India [20]. In addition, the acceptability of using FIs for HIV-related health behaviors has rarely been explored qualitatively [21]. However, the use of FIs remains controversial, with some raising ethical and practical concerns about their use [22].

HPTN 065 evaluated the effect of FIs on linkage to care, defined as attendance at two medical visits after HIV diagnosis. While FIs may serve as an immediate catalyst of behavior change via extrinsic motivation, after the initial decision to accept the FI, the aim is that patients will eventually become intrinsically motivated to engage in long-term HIV care [23]. For this type of FI to work, the behavioral theory of operant conditioning posits that patients must know that they have the potential to earn the reward prior to the desired behavior and then must receive the reward at the time of the behavior [24]. The FI is more likely to be effective if individuals perceive the benefit and receive the benefit sooner rather than later [25]. As such, sites were asked to present the FI for L2C as close as possible to HIV diagnosis.

The HPTN 065 qualitative substudy was developed to explore patient, clinic staff, and site investigator experiences with the use of FIs as part of the HPTN 065 study. This analysis presents patient and provider attitudes and experiences with the L2C FI intervention to assess acceptability with regard to: 1) general opinions about the intervention, 2) agreement with the principle of providing FIs for L2C, and 3) experiences with implementation.

## Methods

### Linkage to care intervention in HPTN 065 study

Nineteen participating HIV test sites (9 in Bronx, 10 in DC) were randomly assigned to provide the FI for L2C from April 2011 to December 2012 in addition to their standard of care linkage services, and 18 HIV test sites were randomly assigned to provide only standard of care. At the FI sites, patients with newly diagnosed HIV or those testing HIV positive but out of care for at least one year were eligible to receive a coupon at the time of their positive HIV test, redeemable within three months at any HIV care site participating in the study (20 in the Bronx, 19 in DC). The coupon could be redeemed for a $25 gift card upon blood draw for CD4 count and viral load tests at the HIV care site, and for a $100 gift card upon meeting with a clinician to review lab results and develop a care plan. The amount of the gift cards was determined based on extensive consultation with the study community advisory group and other stakeholders. During the study, 1,061 coupons were disbursed, and redeemed for 932 $25 and 842 $100 gift cards. Findings from the study showed no statistically significant improvement in the proportion of persons linking from sites that provided the FI coupon compared with those that did not [26]. HPTN 065 is registered on ClinicalTrials.gov as NCT01152918.

### Qualitative substudy design

The qualitative substudy included a convenience sample of patients, staff and site investigators from 21 sites: 9 in the Bronx, NY and 12 in Washington, D.C. Some site investigators and staff members represented multiple sites (Table 1). Participants were from test sites randomized to distribute coupons to patients, as well as care sites that redeemed coupons for gift cards upon linkage; some sites both disbursed coupons and redeemed them for gift cards.

**Table 1:** Summary of sites participating in HPTN 065 qualitative substudy.

Site(s)	Site Type	Test/Care	Envisioned	Patients	Staff	Site
			Implementation^a^			Investigators
**Bronx**						
A	Health center, clinic or organization	test	Sometimes			1
B, C^b^	Hospital	test and care	No		2	1
D	Hospital	test and care	No		2	1
E, F, G^b^	Hospital	test and care	Sometimes	5	2	1
H	Health center, clinic or organization	test and care	Yes			1
I	Health center, clinic or organization	test	Yes			1
**Washington, D.C.**						
J	Health center, clinic or organization	test and care	Yes		1	1
K	Health center, clinic or organization	test	Yes			1
L	Health center, clinic or organization	test and care	Yes			1
M	Hospital (Georgetown)	test and care	Sometimes		1	1
N	Hospital	test and care	Yes	2	1	
O	Health center, clinic or organization	test and care	Yes	4	1	
P	Health center, clinic or organization	test	Yes		1	
Q	Hospital	test and care	Yes			1
R	Health center, clinic or organization	test and care	No		1	
S	Hospital	test and care	Yes		2	1
T	Health center, clinic or organization	test and care	Yes	4	1	1
U	Health center, clinic or organization	test	Yes			1
**Total**				**15**	**15**	**14**

^a^ “Envisioned Implementation” means that the coupon was given to patients immediately following their HIV diagnosis.

^b^ Some hospitals are lumped together because the program was overseen and implemented by the same people at all locations; however, all participants (site investigators, site staff and patients) are counted uniquely in the table.

Data collection and analysis occurred between July and October 2013, after the FI intervention had ended but before HPTN 065 study outcomes were assessed. Semi-structured face-to-face in-depth interviews with patients and focus group discussions with clinic staff members were conducted by trained interviewers from diverse demographic backgrounds. We advertised for and selected individuals with educational and/or practical experience in qualitative research methods and looked for variation in gender, race, ethnicity and sexual orientation. The face-to-face interviewers and focus group facilitators were hired specifically for these activities and did not have any other ties to the research study. Face-to-face interviews were conducted with all patients to maintain confidentiality with regard to their HIV diagnosis. For clinic staff members, focus groups were utilized to gather information from many individuals in a short amount of time, and to allow for cross-talk amongst the sites, because the interventions were incorporated into their standard-of-care somewhat differently. Semi-structured, key informant interviews with site investigators were conducted by phone to maximize participation. The interviewer for the site investigators was part of the overall research project and had a minimal working relationship with each site investigator over the course of the primary study; however, the interviewer had no knowledge of overall study outcomes.

All interviews and focus groups were conducted in English or Spanish, audio-recorded, transcribed and translated, if necessary. Interview and focus group guides (S2–S5 Files), as well as significant training on their use, were provided to all data collectors prior to data collection.

Site staff were asked to identify potential participants using the following eligibility criteria: linked to care with an HPTN 065 coupon during the FI intervention, redeemed their coupon for both the $25 and $100 gift cards; and receiving HIV care at the same clinic where they were linked during the intervention. The fifteen patients (5 in Bronx, 10 in DC) who participated in the interviews were recruited from 4 care sites; all but one had received their coupon at a test site within the same institution. All these participants redeemed a study coupon between January 1, 2012 and March 31, 2013, and were still engaged in care at the time of the interview. Demographic information, clinical data (date of HIV-positive test result, values of first VL and CD4 count upon linkage), and HPTN 065 study data (dates of coupon redemption and receipt of gift cards), were collected for all interview participants from clinic records.

All site investigators were invited to participate in the key informant interviews, of which 14 (6 in Bronx, 8 in DC) agreed; most were physicians, and together, they represented 17 sites.

All financial incentive coordinators and other site staff who distributed coupons and/or gift cards were invited to participate in the focus group discussion. Four focus groups were conducted with a total of 15 staff members (6 in Bronx, 9 in DC) from 11 sites. An average of four staff members participated in each focus group, including FI coordinators, site coordinators, nurses, nurse practitioners, social workers and research or physician assistants, all of whom had an active role in either disbursing coupons or redeeming them for gift cards.

### Data analysis

All transcripts were uploaded into NVivo 10.0 (QSR International) and qualitative thematic content analysis techniques were used to analyze the data, following a process of reading, coding, data display, and reduction [27]. All transcripts from interviews and focus groups were read and initially coded based on questions in the interview guide and emerging themes. Approximately 40% of transcripts were coded by two analysts and manually reviewed to check for inter-coder reliability.

Primary coding reports related to opinions of the intervention, L2C procedures, implementation, and experiences in giving and receiving the FI, were extracted and further analyzed. Emergent sub-themes were codified and applied to data in coding reports. Where applicable, Excel matrices were used to enumerate themes and sub-themes. Memos were developed to summarize findings within each broad theme.

### Ethical considerations

The qualitative substudy protocol was approved by a central IRB (Copernicus Group IRB); the approval included focus groups and investigator interviews. Each site participating in patient interviews was also required to obtain either local IRB approval or approval under the central Copernicus Group IRB submission, depending on their choice or institutional policy. Ten sites were included under the Copernicus Group IRB approval; approval was obtained from the following local IRBs for the remaining 4 sites: Albert Einstein College of Medicine of Yeshiva University IRB, Children’s National Health System IRB, and George Washington University and Medical Center IRB. Written informed consent was obtained from all interview and focus group participants prior to data collection. As the interview with the site investigators were not conducted in person, consent was obtained orally before the phone conversation began. Written documentation was kept to certify that this had been done, and oral consent was recorded audibly. This procedure was included in the study protocol, that underwent IRB review and approval prior to study implementation.

## Results

Staff and site investigators represented a wide range of HIV test and care sites with different ways of implementing the intervention (Table 1). Patient interview participants ranged in age from 24 to 58 years; 80% had newly diagnosed HIV, and 20% had previously diagnosed infection but had been out of care for at least one year (Table 2). Nearly one-third had a CD4 count <200 cells/mL at their initial visit.

**Table 2:** Demographic and clinical data of patient participants.

Patient Characteristics		Total	Total
		(N = 15)	(%)
**Location**	Bronx, NY	5	33
	Washington, D.C.	10	67
**Sex**	Male	10	67
	Female	5	33
**Age**	<26	5	33
	26–45	7	47
	>45	3	20
**Race**	White	1	7
	Black/African American	9	60
	Other	5	33
**Ethnicity**	Hispanic	6	40
	Non-Hispanic	9	60
**Sexual Orientation**	Heterosexual	6	40
	Homosexual	7	47
	Not sure	2	13
**Education**	Did not graduate High School (HS)	4	27
	HS/General Educational Development (GED)	4	27
	>HS/GED	7	46
**Personal Income**	<20,000	12	80
**(USD)**	20,000 to 60,000	3	20
**HIV care status**	Newly diagnosed	12	80
	Re-engaging in care	3	20
**Initial HIV RNA**	<50	2	13
	50–500	1	7
	501–10,000	2	13
**(copies/mL)**	10,001–50,000	4	27
	50,001–100,000	2	13
	>100,001	4	27
**Initial CD4count**	<50	1	7
	50–200	4	27
**(cells/mm**^**3**^**)**	201–500	7	46
	>500	3	20

Qualitative results are described below and summarized in Table 3 by the number of patients, staff, and site investigators discussing each theme, as they relate to the three themes of acceptability examined in this substudy: (1) general opinions of the intervention, (2) the principle of the FI program, and (3) implementation.

**Table 3:** Acceptability of linkage to care: Summary of themes.

	Patients	Staff	Site
			Investigators
	N = 15	N = 15	N = 14
***General opinions***			
**Overall opinion of intervention**			
Positive	13	3	9
Mixed	2	6	0
Negative	0	4	5
**Negative reactions at the time of coupon distribution**	1	6	4
**Amount of the incentive**			
Could be less	0	3	1
Good	7	5	5
Could be more	5	0	0
Should be more	1	0	0
**Financial impact**	8	5	9
***Opinions about the principle of FIs***			
**Concerns about the principle**	7	6	1
**Entitlement^a^**	1	9	0
***Implementation***			
**Implementation challenges**			
None or minor^b^	n/a	3	6
Timing of giving coupons	n/a	8	5
Explaining intervention	n/a	4	1
Staffing	n/a	9	4
Study procedures	n/a	5	4

Note: As these data are derived from open-ended questions, columns may not add up to the total number of participants.

^a^ For staff and site investigators, this category refers to any mention of problems with patients displaying a sense of entitlement, or the development of expectations for receiving gift cards. For patients, this refers to concerns about the development of expectations, or displaying a sense of entitlement in the interview.

^b^ None indicates the staff member of site investigator specifically said there were no implementation challenges. If a staff member or site investigator did not discuss the presence or absence of challenges, they are not included.

### Patient opinions and agreement with the principle of FIs for linkage to care

#### Opinions of the intervention

Patients generally had an overall positive opinion of the FI intervention, and all liked at least some aspect of it. Most commonly, patients were grateful for the funds provided, appreciated the positive element it provided in the context of the negative experience of receiving a diagnosis of HIV, and felt it showed that someone cared about them.

*It's not so much*, *but it's enough for you to know that you're being at least thought of*. *And that should be enough anyway… if nobody else in your life cares that you're positive*, *whomever is behind the funding of that… they care just enough*. *Just somewhat*, *so you know that… you're not entirely alone*. [Patient, non-Hispanic black female, 24 years old]

Additionally, nearly all patients viewed the intervention as beneficial, believing it could encourage linkage to care after an HIV diagnosis, and that gift cards for two visits would be sufficient to encourage patients to become more informed about HIV, begin getting care at a clinic, and then remain in care.

*Honestly it’s a good program*, *it really is*. *And it does work*, *it really does*. *Cause if you*, *if you sit there and you’re already*, *the staff is already super nice and super cool and then you do this*, *go beyond*, *like having different research going on*. *You know you offer this and that*. *It’s like wow*, *they really want you to stay here*. [Patient, Hispanic female, 37 years old]

While opinions of the intervention were generally positive, one patient reported having a negative emotional reaction when he was given the coupon.

*This is going to sound very hard*, *awful but I was like damn I got to get HIV to start getting these benefits… you know I was feeling down*. *So I’m like*, *oh*, *oh my God*, *so now I’m like a charity case or something*. *Or what’s going on*, *why am I getting paid to be sick?* [Patient, Hispanic male, 26 years old]

Despite the initial reaction, the patient reported having no lasting negative perception and felt it was beneficial for encouraging people to link to care.

#### Opinions on the amount and financial impact of the incentive

In general, participants felt that the amount of the FI ($125) was appropriate, but some would have appreciated more. Among patients interviewed, there was agreement that the incentive was useful, whether it allowed them to pay for food or other necessities, transportation to and from the clinic, or prescription and clinic visit co-pays, and many expressed their appreciation of it.

Financial compensation is helping someone, because living with HIV, not being on a clinical trial per se, you still have to pay for medications and doctors’ visits and to be honest with you… I may have used it for transportation here through cab or metro or even to pay for a copay. [Patient, non-Hispanic black male, 24 years old]

#### Philosophical opposition

Despite an overall positive opinion of the program, about half of the patients were opposed to the concept of paying people to link to care and thought people should be self-motivated to link to care for their own health and well-being without an incentive.

*I don't feel that someone should be paid for something that they need to do for themselves…if you don't want to do it*, *that's fine*. *Nobody's going to make you do anything…I don't feel like you should get it if that's the only reason why you're here*. [Patient, non-Hispanic black female, 24 years old]

### Site investigator and staff opinions and agreement with the principle of FIs for linkage to care

#### Site investigator and staff opinions of the intervention

Clinic staff and site investigators had a wider range of opinions than patients about the intervention and were divided between generally positive, generally negative, and mixed opinions (Table 3).

*Personally I think it’s fabulous…if it could do what the point of the study is which is to link them to the care and get them on the medication and do all of that*, *then I think it’s wonderful…Even if we got 10 people over the 2 years who [are] now engaged in care*, *are virally suppressed*, *etc*., *etc*… *That’s 10 more people and that’s invaluable in my mind*. [Staff member]

Site investigators and some staff who expressed negative opinions felt that their clinics already had strong L2C interventions and that an FI could not have an impact or was not necessary.

*I’m not sure it truly led to any significant increase in linkage beyond what we did…Part of that is we have such an active linkage to care methodology in place to start*. *Where you know*, *when folks are tested*, *they’re escorted over personally in to the clinic and into care immediately*. [Investigator]

#### Opinions about the amount of the incentive

Staff and site investigators thought that the amount of the incentive was appropriate or too high; none thought it should have been larger. Several mentioned that it was the highest incentive of any they had seen, or had concerns that the amount would not be sustainable.

*I would say less would be better because A*, *we could be funded for longer and B*, *I feel like it’s*… *showy to be like “I’ve got a $100 for you” for someone who may have never received an amount of $100 ever in their life*. [Staff member]

#### Philosophical opposition

While most site investigators had a positive opinion about the intervention, some strongly opposed the concept.

*It was not a program that I agreed with*. *I didn't think that giving patients financial incentives was an appropriate thing to do…I think it was that patients also have a responsibility towards asking for care*, *and I think that providing the financial incentive…is almost…bribing them without them taking any ownership or responsibility for their own care*. [Investigator]

Staff were more conflicted, while recognizing the financial benefit the FI provided to some patients.

*I was a little conflicted*, *I’m not gonna lie*, *being that I personally know people who are positive and they don’t need incentives to stay in care*. *But I can understand from their point of view*, *why it may help*. [Staff member]

#### Patients’ sense of entitlement

Some staff members, particularly those who interacted with many patients and were most involved in the distribution of gift cards, said the intervention created expectations among patients about being paid to obtain health care, and a sense of entitlement.

*I think they—once they received the $125*, *they expected money to come…for any other services*. *“Oh you want me to stay?* *You’re gonna have to pay me to stay*.*”* [Staff member]

On the other hand, several site investigators discussed initial concern that patients might demand gift cards and develop expectations, but did not see this materialize.

*There were so few incidents of patients really misbehaving during this…and I would have thought that would have been a huge problem*. *That whenever money was involved that people would be demanding things and asking for things and that happened so infrequently*. [Investigator]

#### Sustainability

Staff and site investigators were uncertain about the financial sustainability of FIs for L2C and whether FIs could continue on a large scale.

*I understand the public health rationale*, *but I wonder whether it would be sustainable as public policy*, *because…your taxpayers [would be] like ‘Wait*, *what are we supporting?’* [Staff member]

Others thought it could be justified by the public health benefit.

*These people have HIV*, *and one person with HIV spreads it to everyone*, *it’s not just a contained issue*, *it’s an issue that affects the whole community*. [Staff member]

### Overall interest in future use of FIs for linkage to care

All of the site investigators indicated that they would consider participating in an intervention offering FIs for L2C in the future, even those who expressed negative opinions or did not believe that it would have an effect. They cited the importance of L2C, and were willing to try new ways that could improve the number of patients linking and financially help patients overcome obstacles to initiating HIV care.

*Linkage to care is so important not just for the individual patients*, *but as a public health measure*, *that I think anything we can do to facilitate that is worth it*. *And that quite frankly*, *we are spending so much money on our HIV positive patients that $125 as an FI is a drop in the bucket and money well*, *well spent*. [Investigator]

#### Experience with implementation of the FI intervention

Site investigators and staff described mixed experiences with implementing the FI intervention. Several reported no or only minor challenges.

*It really wasn't a whole lot of work or difficulty organizing it*. *It didn't disrupt the natural flow*, *in other words*, *and was just kind of easily synced into what we were doing anyway…* [Investigator]

However, some staff and site investigators described difficulty associated with the timing of giving coupons, explaining the intervention, and logistical challenges (Table 3).

#### Timing of giving coupons

While HIV test sites were allowed some flexibility to integrate the intervention into their current standard of care, the study required test sites to provide the coupon on the same day as providing the HIV-positive test result to maximize the potential to link to care. However, some staff and site investigators reported that this was not always feasible. Dates reported in coupon data confirm that eight out of the 15 interview participants did not receive the coupon on the same day as their positive test result, instead getting the coupon at a care appointment, after they had already been linked. Staff were sometimes unavailable to give patients the coupon before they left the HIV test site, or to challenges in integrating the coupon disbursement into the visit flow.

*Some patients would be discharged without having gotten the coupon and then we would have to track them down to try to get them the coupon… Patients could be discharged on a Saturday or Sunday and just not have the coupon yet*. *Or we may not have given it to them yet for one reason or another*. [Investigator]

Sometimes staff decided to delay giving coupons to patients who were experiencing emotional turmoil.

*In the beginning*, *I was so focused on encountering and offering the program on the very first day*, *thinking that this was a great motivation thing*. *But then I eventually realized that it’s not the best thing*, *so I went from trying to present the program in the same day*, *to perhaps waiting an appointment or two*, *depending on how the patient was*. [Staff member]

#### Difficulty introducing FI at the time of an HIV diagnosis

Staff members reported that explaining the intervention to patients who had just learned of their HIV infection could be very challenging.

*Having to tell somebody they’re positive really is hard enough without dealing with ‘Can you sign here for this coupon and can you be part of this study?’*. *And the patient is like ‘I just found out that I’m positive*.’ [Staff member]

Some staff suggested that difficulties were exacerbated when study staff who were not especially trained or experienced in counseling patients with newly diagnosed infection were tasked with disbursing the coupons.

*Just telling the patient…about the program was kind of difficult*. *Especially for the other coordinator…he didn’t have the background that I have*. *So it was kind of difficult for him to explain it…He’d gone through the training but what I’m saying is from a social worker standpoint*. [Staff member]

Some site investigators and staff indicated that a small number of patients reacted negatively when the intervention was first described to them.

*The patient is like in tears*, *crying*, *and the worst thing we did is that we started explaining to her [the coupon program]…She didn’t want to hear it*, *she got mad*. *She started crying more*. *She said that we were offending her*. *That number one*, *why were we assuming that she’s not going to come back to her appointment? So we think that she doesn’t care about her health? Is it something about the way she looks?”* [Staff member]

However, most site investigators and staff clarified that most patients did not have negative reactions.

*A lot of patients came to us and actually cried and were thankful for the money*. *And you know*, *the patients that were difficult are very few and [far] between*. [Staff member]

Negative reactions tended to occur more frequently when the FI intervention was first implemented at a site and were sometimes a result of language and cultural misunderstandings. Several clinic staff members and site investigators discussed techniques they employed to minimize negative reactions. Timing of the introduction to the intervention was crucial, as was its introduction by someone with whom the patient was familiar, and being careful to avoid suggesting that the patient would not link to care if they were not offered the incentive.

*I wouldn’t say…that we had as much as an issue*, *with negative reactions to it*. *But again*, *that was a side effect of the fact that by the time we got a patient*, *they [had] probably already spoken with the ID [infectious diseases] social worker*, *or an ID provider*, *who sort of cushioned that…initial reaction; Because going into it we were extremely sensitive to the order in which information was being presented to a patient*, *and really wanted to err on the side of all of the medical issues and all of the*, *you know-emotional issues being dealt with before it got to the point of like ‘Oh and by the way*! *Here’s some money*!*’* [Staff member]

#### Logistical challenges

Both site investigators and staff described some logistical challenges related to the distribution of the FIs, including staff coordination.

*They had to coordinate…we have a lot of tester counselors*, *but only some of them were directly involved in the research…so the research tester counselors had to coordinate with the non-research tester counselors and then they had to also be concerned with the patients that they might've missed*, *who never got the coupon*. [Investigator]

Another challenge was securing coupons and gift cards.

*We needed to have a locked space and because our facilities are so small*, *so we had to have an office that could be locked*, *and we had to have like a little cupboard inside that*. *That may sound so silly to many people*, *but that space is a premium… that took time and effort to have an area that they could keep this*. [Investigator]

Additionally, patients had to retain their coupon after receiving it and present the coupon at the care clinic at both the first and second visit in order to receive the gift cards. Some staff reported problems with patients losing coupons.

*I’ve had people really plead with me between visit 1 and visit 2*. *You’re supposed to give them the coupon*, *they’re supposed to bring it back*. *Now that’s tough for some people*. *That is very*, *very tough*. [Staff member]

## Discussion

We found that the use of FIs for L2C was generally acceptable to patients, investigators, and staff. Patients were grateful for the funds provided, appreciated the positive element it provided at the time of the negative experience of a positive HIV test result, and believed the incentive could encourage others to link to care after their HIV diagnosis. The incentive made patients feel cared for, which enhanced their relationship with their provider, alleviating concerns that using an FI in the context of healthcare can harm the patient-provider relationship that is traditionally built on a foundation of trust [28, 29]. Many patients, staff members, and site investigators agreed that the incentive provided a tangible benefit to patients with financial need, and reported few challenges in administering the FI intervention.

However, our results raise questions that warrant further investigation, and we identified important issues that may have impacted the outcome of the HPTN 065 L2C FI intervention, which did not show an increase in linkage to care rates [26], and may have implications for the feasibility and effectiveness of future FI interventions. Despite overall positive acceptability of the FI intervention and even a desire to participate in FI interventions in the future, concerns remain about paying people for desirable health behaviors, the sustainability of FI interventions, fears that FIs might foster unrealistic expectations among FI recipients, and the belief that FIs could not add anything to already existing and strong L2C programs. These types of concerns about the use of health-promoting financial incentives are not unique and have been previously cited in the literature [21, 30–34].

Additionally, the timing that was theorized for maximum impact of the incentive (i.e. immediately after a new HIV diagnosis) and how the coupon was presented posed challenges for sites, and as a result the intervention was not always implemented as designed. Based on the behavioral theories cited earlier in this paper, timing of the FI is important for maximizing its effectiveness. In the HPTN 065 study, it was envisioned that patients would receive the FI coupon immediately after learning their positive HIV test result. However, our findings indicate that sites found it difficult to present the coupon at the time of the HIV diagnosis. Sensitivity to patients’ medical and emotional needs was the first priority, and staff found it necessary to tailor or vary the timing of FI coupon distribution.

The way the FI is presented can also affect the acceptability of the FI and may have reduced its effectiveness. As decisions are often affected by one’s emotional state [35], a challenge lies in presenting the intervention in such a way as not to disturb or disrupt this state, which is the very fragile time of receiving an HIV infection diagnosis, so that the patient remains receptive to the idea of linkage to care, and so that the patient-provider relationship is not damaged. We found that some patients perceived the offer of the FI as condescending, trivializing, or patronizing–implying that they would not seek HIV care without an inducement. Staff training on how to explain the FI with an emphasis on consideration for the delicate nature and the timing of the intervention will prove critical for the introduction of any future FI for L2C interventions.

### Strengths and limitations

This qualitative substudy allowed for exploration of a range of themes relating to the acceptability and implementation of FIs for L2C through comparing and contrasting the experiences of patients, a variety of staff, and site investigators from multiple study sites. However, the substudy also has some limitations. The nonrandom selection of participants and small sample size limit the generalizability of the findings. In particular, patients were selected for interviews by staff at their HIV care sites, and staff might have recruited patients with whom they had a good relationship or who had a more positive opinion of the intervention. Because of the substudy design, it was only possible to interview patients who successfully linked with the use of a coupon and were currently engaged in HIV care. In addition, the parent study did not track individuals who were offered a coupon, but declined it. Patients who received the coupon but did not link to care, or who declined to receive it in the first place, might have very differing views on the acceptability of FI intervention. The qualitative substudy was conducted before the effectiveness outcome was determined. Some substudy participants might have expressed different opinions if they had known that the FIs did not have a significant effect on linkage to care rates.

Researchers have increasingly been exploring the use of financial incentives as a potential means to encourage patients’ positive health behaviors and decisions. However, few studies have examined FIs among persons disbursing and receiving FIs [30]. Our findings can help interpret the results of HPTN 065, and inform future design and implementation of FI interventions for L2C and other health-related behaviors.

## Conclusion

Patients, clinic staff and site investigators in the HPTN 065 study had overall favorable views on the use of FIs for L2C. While HPTN 065 was not able to demonstrate effectiveness of FIs for L2C, these perspectives from patients and staff provide important insights into the way the study was carried out and unanticipated factors that might influence the acceptability and effectiveness of FIs. These will be useful for the design and implementation of future studies. Future efforts to assess the effectiveness of FIs for L2C need to consider novel ways to integrate the intervention within existing procedures, to consider optimal timing and presentation of the FI, and will need to address lingering philosophical and ethical concerns about the use of FIs for health behavior change.

## Supporting information

S1 File Consolidated Criteria for Reporting Qualitative Studies (COREQ): 32-item checklist. (PDF)

S2 File Patient in-depth interview guide. (PDF)

S3 File Focus group guide for L2C test sites. (PDF)

S4 File Focus group guide for L2C care sites. (PDF)

S5 File Site investigator interview guide. (PDF)
