# Supplementary material for: It’s all in the timing: Acceptability of a financial incentive intervention for linkage to HIV care in the HPTN 065 (TLC-Plus) study
Source: PLoS One. 2018 Feb 2;13(2):e0191638. doi: 10.1371/journal.pone.0191638 (PMC5796687; doi:10.1371/journal.pone.0191638)
Supplement: S1 File — (PDF) [file pone.0191638.s001.pdf]

Consolidated criteria for reporting qualitative studies (COREQ): 32-item checklist for:

It's All in the Timing: Acceptability of a Financial Incentive Intervention for Linkage to HIV Care in the HPTN 065 (TLC-Plus)

Domain 1: Research Team and Reflexivity:

<u>Personal Characteristics</u>

1. Interviewer/facilitator: Which author/s conducted the interview or focus group?

   Semi-structured face-to-face in-depth interviews with patients and focus group discussions with clinic staff members were conducted by trained interviewers from diverse demographic backgrounds. Key informant telephone interviews with site investigators were conducted by one interviewer, a member of the protocol team who had no knowledge of overall study outcomes but had a minimal working relationship with the investigators over the course of the HPTN 065 study. All patient interviews and staff focus groups were conducted by non-authors listed in Acknowledgements section of the paper. Theresa Gamble, PhD, conducted all interviews with the site investigators by phone.

2. Credentials: What were the researcher's credentials? E.g. PhD, MD

   The author's credentials are as follows:

   - Victoria Shelus (VS), MEM
   - Jamilah Taylor (JT), BA
   - Elizabeth Greene (EG), MPH
   - Jill Stanton (JS), BA
   - Allison Pack (AP), MPH
   - Elizabeth Tolley (ET), PhD
   - Bernard Branson (BB), MD
   - Wafaa El-Sadr (WES), MD, MPH
   - June Pollydore (JP) (none)
   - Theresa Gamble (TG), PhD

   All non-author data collectors held Master's level degrees or above.

3. Occupation: What was their occupation at the time of the study?

   TG, EG, ET, VS, JT, JS, BB and WES were (and all except BB still are) full time researchers with many combined years of experience in the HIV prevention field. JP was a site investigator at one of the HPTN 065 study sites. All non-author data collectors were hired and trained specifically to conduct the qualitative data collection for this study.

4. Gender: Was the researcher male or female?

   The author's identified genders are as follows:

   - Victoria Shelus (VS), female
   - Jamilah Taylor (JT), female
   - Elizabeth Greene (EG), female
   - Jill Stanton (JS), female
   - Allison Pack (AP), female
   - Elizabeth Tolley (ET), female
   - Bernard Branson (BB), male
   - Wafaa El-Sadr (WES), female
   - June Pollydore (JP), female
   - Theresa Gamble (TG), female

   Six of the non-author data collectors identified as female and one as male.

5. Experience and training: What experience or training did the researcher have?

   - TG, BB and WES: Extensive quantitative and clinical research experience in HIV, exposure to qualitative methodology
   - ET: Extensive qualitative research training and experience in multiple health fields
   - EG AP: Theoretical and practical training and experience with qualitative research methods
   - VS, JS and JP: Exposure to qualitative methodology

   All non-author data collectors were selected and hired on the basis of prior qualitative research and data collection experience, as well as experience working with HIV-infected and diverse populations.

<u>Relationship with Participants</u>

6. Relationship established: Was a relationship established prior to study commencement?

   No relationship was established between study participants and the interviewers/focus group facilitators except for the site investigator interviews. TG had a minimal working relationship with the site investigators prior to their interviews.

<u>Participant Knowledge of the Interviewer</u>

7. What did the participants know about the researcher? e.g. personal goals, reasons for doing the research

   Nothing, except for the site investigators, who were aware of their interviewer's reasons for conducting the research due to their working relationship with her.

8. Interviewer characteristics: What characteristics were reported about the interviewer/facilitator? e.g. Bias, assumptions, reasons and interests in the research topic

   The face-to-face interviewers and focus group facilitators were hired specifically for these activities and did not have any other ties to the research study. We advertised for and selected individuals with either educational or practical experience in qualitative research methods and looked for variation in gender, race, ethnicity and sexual orientation. The interviewer for the site investigators was part of the overall research project and had a minimal working relationship with each one over the course of the primary study. No one was aware of the outcome of the FI intervention when the qualitative data were collected. We do not believe that there were any biases of significance to report, except for the interviewer for the site investigators, who were fully aware of the potential bias at the time.

Domain 2: Study Design

Theoretical framework

Methodological Orientation and Theory

9. What methodological orientation was stated to underpin the study? e.g. grounded theory, discourse analysis, ethnography, phenomenology, content analysis

   Our study explored individual patient and provider attitudes towards and experiences with FI the intervention for linkage-to-care, including how the FI influenced motivation to seek care or choice of care sites. Qualitative applied thematic content analysis techniques were used to analyze the data, following a process of reading, coding, data display, and reduction, as described in chapter 6 of Qualitative Methods in Public Health (Ulin et al. 2005 1st ed, Tolley et al. 2016 2nd ed.).

10. Sampling: How were participants selected? e.g. purposive, convenience, consecutive, snowball

   The qualitative substudy included a convenience sample of patients, staff, and site investigators from 21 sites: 9 in the Bronx, NY and 12 in Washington, D.C. Participants were from test sites randomized to distribute coupons to patients, as well as care sites that redeemed coupons for gift cards upon linkage; some sites both disbursed coupons and redeemed them for gift cards. HPTN 065 test and care sites that agreed and were able to obtain IRB approval prior to study implementation formed the subset of sites from which participants were recruited. All patients interviewed must have redeemed a study coupon between January 1, 2012 and March 31, 2013, and be still engaged in care at the time of the interview.

11. Method of approach: How were participants approached? e.g. face-to-face, telephone, mail, email

    The site investigators (for interviews) and staff (for focus groups) were all approached via email by members of the study team; sites were responsible for the recruitment of participants at their site, with primarily face-to-face and telephone contact being used for recruitment. Sites were provided with talking points for approaching patients but utilized their own recruitment strategies.

12. Sample size: How many participants were in the study?

    Fifteen patients (5 in Bronx, 10 in DC) participated in the interviews were recruited from 4 care sites; all but one had received their coupon at a test site within the same institution. Key informant interviews were conducted with 14 site investigators (6 in Bronx, 8 in DC), who were mostly physicians, representing 17 sites. Four focus groups were conducted with a total of 15 staff members (6 in Bronx, 9 in DC) from 11 sites.

13. Non-participation: How many people refused to participate or dropped out? Reasons?

    We did not track refusals. Because the study was cross-sectional (interviewed at only one timepoint), there were no drop outs.

<u>Setting</u>

14. Setting of data collection: Where were the data collected? e.g. home, clinic, workplace

    Site investigator interviews were conducted by phone, with both the interviewer and interviewee at their place of work. Individual interviews took place at the clinic where they normally received their HIV care. Focus groups took place in a variety of locations that had meeting venues convenient to participants.

15. Presence of non-participants: Was anyone else present besides the participants and researchers?

    No non-participants were present during individual interviews or focus groups.

16. Description of sample: What are the important characteristics of the sample? e.g. demographic data, date

    As this manuscript focused on the acceptability of using a financial incentive for linkage-to-care, we chose participants who had received or distributed the financial incentive. Within that large category, we sought diversity by including a range of sites in our sample. No other criteria were used. The table below shows the demographic and clinical data for the participants.

Demographic and Clinical Data of Patient Participants

Patient Characteristics		Total (N=15)	Total (%)
**Location**	Bronx, NY<br>Washington, D.C.	5<br>10	33%<br>66%
**Sex**	Male<br>Female	10<br>5	66%<br>33%
**Age**	<26<br>26 – 45<br>>45	5<br>7<br>3	33%<br>46%<br>20%
**Race**	White<br>Black/African American<br>Other	1<br>9<br>5	6%<br>66%<br>33%
**Ethnicity**	Hispanic<br>Non-Hispanic	6<br>9	40%<br>60%
**Sexual Orientation**	Heterosexual<br>Homosexual<br>Not sure	6<br>7<br>2	40%<br>46%<br>13%
**Education**	Did not graduate High School (HS)<br>HS/General Educational Development (GED)<br>>HS/GED	4<br>4<br>7	26%<br>26%<br>46%
**Personal Income (USD)**	<20,000<br>20,000 to 60,000	12<br>3	80%<br>20%
**HIV care status**	Newly diagnosed<br>Re-engaging in care	12<br>3	80%<br>20%
**Initial HIV RNA (copies/mL)**	<50<br>50 – 500<br>501 - 10,000<br>10,001 - 50,000<br>50,001 – 100,000<br>>100,001	2<br>1<br>2<br>4<br>2<br>4	13%<br>6%<br>13%<br>26%<br>13%<br>26%
**Initial CD4 count (cells/mm^3)**	<50<br>50 – 200<br>201 – 500<br>>500	1<br>4<br>7<br>3	6%<br>26%<br>46%<br>20%

Data Collection

17. Interview guide: Were questions, prompts, guides provided by the authors? Was it pilot tested?

    Interview and focus group guides, as well as significant training, were provided to all data collectors. The guides were not pilot tested with participants nor shared with participants prior to the interview or focus group; however, they were tested, and subsequently revised, during the interviewer training process. Interview guides have been provided as supplemental data.

18. Repeat interview: Were repeat interviews carried out? If yes, how many?

    No repeat interviews were conducted.

19. Audio/visual recordings: Did the research use audio or visual recording to collect the data?

    All interviews and focus groups were audio recorded; none were visually recorded.

20. Field notes: Were field notes made during and/or after the interview or focus group?

    Because all data collection was audio-recorded, field notes were kept to a minimum for the individual interviews; however, extensive notes were taken during the focus group discussion during the sessions by a second facilitator/data collector.

21. Duration: What was the duration of the interviews or focus group?

    Each interview was roughly one hour in length and the focus groups varied between one to two hours.

22. Data saturation: Was data saturation discussed?

    Our protocol included a target number of interviews/FGDs per each category of participant. These targets were determined with the concept of saturation in mind. Consequently, we did not assess saturation in an on-going manner (with the aim of modifying our sampling approach as might be done in a study guided by grounded theory.)

23. Transcripts returned: Were transcripts returned to participants for comment and/or correction?

    No transcripts were returned to participants for comment or correction.

Domain 3: Analysis and Findings

<u>Data analysis</u>

24. Number of data coders: How many data coders coded the data?

    VS, JT, EG, JS, AP, ET and TG all served as data coders for the project.

25. Description of the coding tree: Did authors provide a description of the coding tree?

    All transcripts from interviews and focus groups were read and initially coded based on questions in the interview guide and emerging themes using the qualitative data analysis software NVivo 10 (QSR International). After the initial codebook was developed, approximately 40% of transcripts were coded by two analysts and manually reviewed to check for inter-coder reliability. An iterative process was used to address discrepancies, agree upon interpretation, and further clarify the codebook and coding of transcripts.

26. Derivation of themes: Were themes identified in advance or derived from the data?

    All transcripts from interviews and focus groups were read and initially coded based on questions in the interview guide and emerging themes using the qualitative data analysis software NVivo 10 (QSR International). After the initial codebook was developed, approximately 40% of transcripts were coded by two analysts and manually reviewed to check for inter-coder reliability. An iterative process was used to address discrepancies, agree upon interpretation, and further clarify the codebook and coding of transcripts. Primary coding reports related to opinions of the intervention, L2C procedures, implementation, and experiences in giving and receiving the FI, were extracted and further analyzed. Emergent sub-themes were codified and applied to data in coding reports. Where applicable, Excel matrices were used to enumerate themes and sub-themes. Memos were developed to summarize findings within each broad theme.

27. Software: What software, if applicable, was used to manage the data?

    The team used the qualitative data analysis software NVivo 10 (QSR International) to manage the data and conduct analysis.

28. Participant checking: Did participants provide feedback on the findings?

    No participants provided feedback on the findings, except for three site investigators who reviewed the manuscript.

<u>Reporting</u>

29. Quotations presented: Were participant quotations presented to illustrate the themes / findings? Was each quotation identified? e.g. participant number

    Yes, quotes were used, and they were identified by study role, and for participants, age, race, ethnicity and gender.

30. Data and findings consistent: Was there consistency between the data presented and the findings?

    Yes.

31. Clarity of major themes: Were major themes clearly presented in the findings?

    Yes, the manuscript is organized clearly by theme.

32. Clarity of minor themes: Is there a description of diverse cases or discussion of minor themes?

    Yes, minor themes are discussed in the manuscript.
