# Supplementary material for: It’s all in the timing: Acceptability of a financial incentive intervention for linkage to HIV care in the HPTN 065 (TLC-Plus) study
Source: PLoS One. 2018 Feb 2;13(2):e0191638. doi: 10.1371/journal.pone.0191638 (PMC5796687; doi:10.1371/journal.pone.0191638)
Supplement: S2 File — (PDF) [file pone.0191638.s002.pdf]

HPTN 065 Qualitative Substudy

Patient In-depth Interview Guide for L2C with FIs

<u>TURN ON YOUR AUDIO RECORDER.</u> *Read the PID, date, site, and your name into the recorder.*

Thank you again for agreeing to speak with me today. The purpose of these interviews is to understand the patient experiences and views of the coupon program for linking into HIV care.

As a reminder, participating in this interview is your choice. Someone at this clinic should have talked to you about this study and asked you to sign an informed consent form. Can you please confirm that you have voluntarily agreed to participate and signed the form?

I'd also like to remind you that this interview is being recorded. This is so that we can talk and I don't have to stop you while I take notes. We will destroy the recording when the study is complete. Can you please confirm that you have agreed to have this interview recorded?

During the interview, let me know if there are any questions that you do not feel comfortable answering, and we can move on. You may choose to answer a question or not, or to stop at any time.

I will do everything possible to keep your information private, and I will not discuss your answers with anyone. You have been assigned an ID number so that your name will not be used.

Do you have any questions before we get started?

I want to clarify the language I'll be using today. During this interview, I'm going to ask you some questions about your opinions of the linkage-to-care coupon program. From now on, when I talk about the coupon program, I'm talking about the program where patients got a coupon after testing HIV positive that they could redeem for gift cards when they went to an HIV care clinic.

[Note to Interviewer: Show participant laminated picture of coupon and gift cards]

<u>I. Ongoing HIV Care</u>
I first want to talk about your visits to this clinic for HIV care.

1. Can you tell me some reasons why you come to your HIV care appointments?
    a. How often do you come to HIV care appointments?
    b. What happens during these appointments?
    c. How have your reasons changed since you first started in care?

2. What are some things that make it hard to regularly come to your HIV care appointments?
    a. How do you overcome these challenges?

3. What are some things that make it easier to regularly come to your HIV care appointments?

II. Linking-to-Care
*[*Remember: this may not have been the first time this patient has linked to HIV care.]*

4. Can you tell me what your linkage experience was like? What I mean by that is your experience after getting your HIV-positive test result and then coming to this clinic for HIV care for the first time.
    a. Where did you get your HIV test?
    b. How did the person who tested you or the place where you were tested help you get here?

5. Why did you decide to come to HIV care after your HIV-positive test?
    a. Was this the first time you tested HIV-positive?
    b. Was this the first time you saw a doctor for HIV care?
    c. How long did it take you to make the decision to get HIV care?
    d. Did anyone help you make that decision?

6. How did you decide to come to this particular clinic for HIV care?
    a. Would you have chosen a different care clinic if you could not have gotten the $25 and $100 gift cards?

7. What are some reasons why it may have been difficult to go to your HIV care appointment for the first time after your HIV-positive test?
    a. How did you overcome these challenges?
    b. How long after your HIV-positive test did you go for your first care appointment?

8. What are some things that may have made it easier for you to go to your first HIV care appointment after your HIV-positive test?

III. Experience with and understanding of the coupon program
I want to now talk more about your experiences with the coupon program for linkage-to-care.

9. What was your understanding about how the coupon program for linkage-to-care worked?
    a. What about the gift cards – what was your understanding of what they were for in this program? *[Note: probe for both $25 and $100 gift card]*

10. Thinking back to when you were offered the coupon, how was it explained to you?
    a. Who gave you the coupon?
    b. When did they give you the coupon?
    c. How did they explain what would happen when you got to the care clinic?

11. How did you feel when you were offered the coupon?

12. How long after you got the coupon did you redeem it for the first gift card?
    a. What about the second gift card?

13. How did you feel when you redeemed your coupon for the gift cards at your care clinic?

14. Can you tell me about how you used your gift cards?
    a. Why did you decide to use your gift cards that way?

IV. Opinions of the coupon program

15. What did you think about this coupon program for linkage-to-care?
    a. What did you like about it?
    b. What did you dislike about it?

16. In general, what do you think about the idea of paying people to encourage them to link to HIV care after an HIV-positive test?
    a. What do you think the benefits of such a program are?
    b. What concerns do you have about such a program?

17. What do you think about the amount of the gift cards ($25 and $100)?

V. Community awareness and final thoughts about the coupon program

18. When you were offered the coupon, was that the first time you had heard about the program?
    a. If no, probe for how they heard about it, what they heard, and who they heard it from.

19. What do you think people in your community have heard about the coupon program?
    a. How do you think they knew about it?

20. Do you have any final thoughts or comments about the coupon program to encourage people to get into HIV care?

Before we finish our conversation today, I have a few questions about your background, family, and education. Having this information about all of the participants in our study will help us with our research, but you don't have to answer any questions that make you feel uncomfortable.

[ADMINISTER DEMOGRAPHIC QUESTIONNAIRE]

Those are all the questions that I have for you today. Thank you again for coming to talk with me and sharing your thoughts and experiences. As you know, you will receive a $50 for participating in this study, so I'll take you to check in with the staff at the clinic, who will give you the $50.
