# Supplementary material for: It’s all in the timing: Acceptability of a financial incentive intervention for linkage to HIV care in the HPTN 065 (TLC-Plus) study
Source: PLoS One. 2018 Feb 2;13(2):e0191638. doi: 10.1371/journal.pone.0191638 (PMC5796687; doi:10.1371/journal.pone.0191638)
Supplement: S3 File — (PDF) [file pone.0191638.s003.pdf]

HPTN 065 Qualitative Substudy
FOCUS GROUP GUIDE FOR LINKAGE-TO-CARE FI TEST SITES

Administrative Questions:

FG ID: [___|___|___|___] **Date: ______________________________**

Facilitator: _________________________ **Note Taker:_________________________**

Number of Participants: ______________

Informed Consent Obtained from All Participants? ☐ **YES** ☐**NO**

Start Time of Focus Group: ___________

End Time of Focus Group: ____________

BEFORE THE FOCUS GROUP STARTS:

- ☐ All participants should sign in on the sign-in sheet. File completed sign-in sheet in your study folder.
- ☐ Give everyone a number name tag and ask them to display it on their shirt somewhere visible.
- ☐ Provide two (2) copies of the consent forms to all participants, allow them time to read the forms completely, have them sign both forms. Give the participant one copy, keep one copy in your study folder.
- ☐ Allow participants time to get any drinks/snacks and settle in.

TURN ON YOUR AUDIO RECORDER. *Read the FG ID, date, and your name into the recorder.*

Thank you again for agreeing to participate in this focus group today. The purpose of the focus group is to better understand the experiences you had with implementing the linkage-to-care coupon program at your test site over the past two years, and what you thought about the program. This information will help us better interpret the results of the HPTN 065 (or TLC-Plus) study, and will be useful in developing interventions like this one in the future.

As a reminder, your participation in this focus group is voluntary. If there is a question you do not feel comfortable answering, you do not need to answer it. Your responses in this focus group will be kept confidential. We will not use your name during the discussion, and we ask that when referring to each other, you please use the number on each other's name tags rather than names.

This focus group is being audio recorded. Before we begin, I'd like to confirm that you have given your voluntary consent for this focus group to be recorded. Please say or indicate that you have given your consent for this. ***[FACILITATOR: Confirm verbally that everyone in the room has given consent.]***

Before we get started, I want to clarify the language I will be using today. When I talk about the coupon program, I am referring to the linkage-to-care coupons that you gave to clients who tested HIV-positive, to help them link into HIV care. Does anyone have any questions before we get started?

[Section 1: Professional view of coupon implementation at clinic]

I'd like to start by talking a little bit about your professional opinion about the way that the coupon program was implemented at your clinic.

1. Please think back on how the coupon program was implemented at your site. What were some of the major challenges in implementing the program?
   a. How were these challenges overcome?

2. How did the coupon program change how you talk to clients testing positive about linkage-to-care?

[Section 2: Professional view of how clients perceived the coupon program.]

Now let's talk about how you think that the clients at your clinic may have viewed the coupon program.

3. In general, what do you think your clients liked and disliked about the coupon program?
   a. If not mentioned, probe for what they liked about it.
   b. If not mentioned, probe for what they disliked about it.

4. What did clients think the coupon was for?

5. In general, what were the reactions of clients when you gave them the coupons?
   a. How did these reactions vary?

6. Generally speaking, how often did you have clients who refused to accept the coupon?
   a. Why did these clients say they did not want the coupons?

[Section 3: Perceptions of coupons' impact on client behavior]

I want to talk now about the impact that you think that the coupon program may or may not have had on clients' behavior. Please keep in mind that we don't know the results of the study yet, so please answer these questions based on your own experiences with the coupons and with the clients at your clinic.

7. In your opinion, how might the coupon have changed client behavior, for better or for worse?
   a. Why do you think this?

8. For what types of clients do you think the coupon may have worked the best?
   a. Why do you think this?

[Section 4: Perception about community awareness and possible migration efforts.]

When this study first started, there was a lot of concern in the community about the potential for site migration if clients started to learn which clinics were giving out the gift cards. We are looking at this in surveillance data, but we don't know the outcome yet. We want to talk to you a little bit about your perception of this issue from the perspective of your clinic.

9. Do you think that people in the community knew about the coupon program?
    a. What makes you think this?

10. Did you have clients who sought testing at your clinic specifically because they knew about the linkage-to-care coupons?
    a. If yes, how do you know this?
    b. If yes, was this a problem for your clinic?

[Section 5: Personal opinions about coupon program.]

Now that we've discussed your professional views about the program's implementation and how you think it was perceived by clients, I'd like to talk about your personal opinions about the coupon program.

11. In general, what did you like and dislike about the coupon program?
    a. If not mentioned, probe for what they liked about it.
    b. If not mentioned, probe for what they disliked about it.

12. How did you feel when you gave clients the coupons?
    a. How did your response vary in different situations or with different clients?

13. As the coupon program lasted for about 2 years at your site, can you tell me a little about how your opinion of the program has changed over time?

14. In your opinion, what did you think of the amount of the linkage-to-care coupon ($25 and $100)?
    a. Why did you think that?

15. In general, what do you think about the idea of giving people financial incentives in an effort to encourage them to link to care?
    a. What do you think the benefits of such a program are?
    b. What concerns do you have about such a program?

16. Before we end, do you have any additional thoughts about the coupon program that you'd like to share with us?

That concludes our focus group. Thank you again for coming here today to share your thoughts and experiences with us. As you know, you will receive $50 for participating in this focus group. The study team at FHI 360 will mail you your gift card tomorrow and you should receive it soon.
