# Supplementary material for: It’s all in the timing: Acceptability of a financial incentive intervention for linkage to HIV care in the HPTN 065 (TLC-Plus) study
Source: PLoS One. 2018 Feb 2;13(2):e0191638. doi: 10.1371/journal.pone.0191638 (PMC5796687; doi:10.1371/journal.pone.0191638)
Supplement: S4 File — (PDF) [file pone.0191638.s004.pdf]

HPTN 065 Qualitative Substudy
FOCUS GROUP GUIDE FOR LINKAGE-TO-CARE CARE SITES

**Administrative Questions:**	
**FG ID: [___|___|___|___]**	**Date: ______________________________**
**Facilitator: _________________________**	**Note Taker:_________________________**
**Number of Participants: ______________**	
**Informed Consent Obtained from All Participants? ☐ YES ☐NO**	
**Start Time of Focus Group: ___________**	
**End Time of Focus Group: ____________**	

BEFORE THE FOCUS GROUP STARTS:

- ☐ All participants should sign in on the sign-in sheet. File completed sign-in sheet in your study folder.
- ☐ Give everyone a number name tag and ask them to display it on their shirt somewhere visible.
- ☐ Provide two (2) copies of the consent forms to all participants, allow them time to read the forms completely, have them sign both forms. Give the participant one copy, keep one copy in your study folder.
- ☐ Allow participants time to get any drinks/snacks and settle in.

TURN ON YOUR AUDIO RECORDER. *Read the FG ID, date, and your name into the recorder.*

Thank you again for agreeing to participate in this focus group today. The purpose of the focus group is to better understand the experiences you had with implementing the linkage-to-care financial incentive program at your clinic over the past two years, and what you thought about the program. This information will help us better interpret the results of the HPTN 065 (or TLC-Plus) study, and will be useful in developing interventions like this one in the future.

As a reminder, your participation in this focus group is voluntary. If there is a question you do not feel comfortable answering, you do not need to answer it. Your responses in this focus group will be kept confidential. We will not use your name during the discussion, and we ask that when referring to each other, you please use the number on each other's name tags rather than names.

This focus group is being audio recorded. Before we begin, I'd like to confirm that you have given your voluntary consent for this focus group to be recorded. Please say or indicate that you have given your consent for this. ***[FACILITATOR: Confirm verbally that everyone in the room has given consent.]***

Before we get started, I want to clarify the language I will be using today. When I talk about the FI – or "financial incentive" - program today, I'm referring to the $25 and $100 gift cards that you gave patients who linked to care with HPTN 065 coupons. As you may know, there was another gift card program that involved $70 gift cards for viral suppression, but today we are only going to be talking about the $25 and $100 gift cards for linkage-to-care. Does anyone have any questions before we get started?

[Section 1: Professional view of gift card implementation at clinic]

I'd like to start by talking a little bit about your professional opinion about the way that the FI program for linkage-to-care was implemented at your clinic.

1. Please think back on how the FI program was implemented at your site. What were some of the major challenges in implementing the program?
    a. How were these challenges overcome?

[Section 2: Professional view of how clinic patients perceived the gift card program.]

Now let's talk about how you think that the patients at your clinic may have viewed the FI program for linkage-to-care.

2. In general, what do you think your patients liked and disliked about the FI program for linkage-to-care?
   a. If not mentioned, probe for what they liked about it.
   b. If not mentioned, probe for what they disliked about it.

3. What did the patients think the $25 and $100 gift cards were for?

4. In general, what were the reactions of patients when you gave them the gift cards?
   a. How did these reactions vary?

[Section 3: Perceptions of gift cards' impact on patient behavior]

I want to talk now about the impact that you think that the FI program may or may not have had on patients' linkage-to-care behavior. Please keep in mind that we don't know the results of the study yet, so please answer these questions based on your own experiences with the gift cards and with the patients at your clinic.

5. In your opinion, how might the linkage-to-care coupon and gift cards have changed patient behavior, for better or for worse?
   a. Can you give me some examples?

6. For what types of patients do you think the linkage-to-care coupon and gift cards may have worked the best?
   a. Why do you think this?

[Section 4: Perception about community awareness and possible migration efforts.]

When this study first started, there was a lot of concern in the community about the potential for site migration if patients started to learn which clinics were giving out the gift cards. We are looking at this in surveillance data, but we don't know the outcome yet. We want to talk to you a little bit about your perception of this issue from the perspective of your clinic.

7. Do you think that people in the community knew about the FI program for linkage-to-care?

    a. What makes you think this?

8. Did you experience new patients requesting a linkage-to-care gift card even though they never received a linkage-to-care coupon? If so, why do you think this happened?

[Section 5: Personal opinions about gift card program.]

Now that we've discussed your professional views about the program's implementation and how you think it was perceived by patients I'd like to talk about your personal opinions about the FI program for linkage-to-care.

9. In general, what did you like and dislike about the FI program for linkage-to-care?
    a. If not mentioned, probe for what they liked about it.
    b. If not mentioned, probe for what they disliked about it.

10. How did you feel when you gave patients the gift cards for redeeming their coupon?
    a. How did your response vary in different situations or with different patients?

11. As the linkage-to-care FI program lasted for about 2 years at your sites, can you tell me how your opinion of the program changed over time?

12. In general, what do you think about the idea of giving people financial incentives in an effort to encourage them to link to care?
    a. What do you think the benefits of such a program are?
    b. What concerns do you have about such a program?

13. In your opinion, what did you think of the amount of the gift cards ($25 and $100)?
    a. Why did you think that?

14. Before we end, do you have any additional thoughts about the linkage-to-care FI program that you'd like to share with us?

That concludes our focus group. Thank you again for coming here today to share your thoughts and experiences with us. As you know, you will receive $50 for participating in this focus group. The study team at FHI 360 will mail you your gift card tomorrow and you should receive it soon.
