# Supplementary material for: It’s all in the timing: Acceptability of a financial incentive intervention for linkage to HIV care in the HPTN 065 (TLC-Plus) study
Source: PLoS One. 2018 Feb 2;13(2):e0191638. doi: 10.1371/journal.pone.0191638 (PMC5796687; doi:10.1371/journal.pone.0191638)
Supplement: S5 File — (PDF) [file pone.0191638.s005.pdf]

HPTN 065 Qualitative Substudy
Investigator of Record (IoR) / Site Investigator Interview Guide

Care Site- Redeemed L2C coupons

IoR Opinions of the FI Program

- Overall, what do you think about the FI program for linkage-to-care?
    - What did you like and dislike about it?
- Did your opinion of the program change over time, and if so, how?

Impact of the FI Program on the Clinic

- What challenges did you or your staff face in implementing the FI program for linkage-to-care?
- How did the FI program for linkage-to-care change the environment in your clinic, if at all?
- How aware of the FI program for linkage-to-care were members of your staff?

Impact of the FI Program on Patients

- In your opinion, what was the impact of the FI program for linkage-to-care on the patients at your clinic?
    - What do you think were the benefits of the program for your patients?
    - What do you think were the drawbacks of the program for your patients?
    - What were the differences among patients in how they reacted to the program?

Interactions with Patients during the FI Program

- How did you or your staff describe the FI program ($25/$100 gift cards) for linkage-to-care to your patients?
    - What do you think your patients understood about why they were receiving the gift cards?
    - Do you think your patients made the connection between linking to care, their health and the gift cards? Why or why not?
- How did the FI program change the way you or your staff interacted with your patients?
- How did the FI program change the way you or your staff talked to your patients about linkage-to-care?

End of the FI Program

- How do you think the end of the FI program for linkage-to-care has or will impact your clinic?
- If you were given the opportunity to offer FIs to patients for linkage-to-care in the future, would you do so? Why or why not?
- Is there anything about the FI program for linkage-to-care that you would change?

Site Migration

- How well do you think that people in the community knew about the FI program for linkage-to-care?
- Are you aware of any situations where individuals who were ineligible for the FI program for linkage-to-care tried to redeem coupons for gift cards? If so, can you give some examples?

Do you have any additional thoughts about the FI program for linkage-to-care that you'd like to share with us?

Care Site- Did not redeem L2C coupons
(Note: these questions will only be asked to FI Test/SOC Care sites or FI Care sites)

IoR Opinions of the FI Program

- Although your site never redeemed any coupons for gift cards as part of the FI intervention for linkage-to-care, do you have any opinions about the intervention?
- If you were given the opportunity to offer FIs to patients for linkage-to-care in the future, would you do so? Why or why not?

Site Migration

- How well do you think that people in the community knew about the FI program for linkage-to-care?

Do you have any additional thoughts about the FI program for linkage-to-care that you'd like to share with us?

FI Test Site

IOR Opinions of the FI Program

- Overall, what did you think about the FI program for linkage-to-care?
    - What did you like and dislike about it?
- Did your opinion of the program change over time, and if so, how?

Impact of the FI Program at your Site

- What challenges did you or your staff face in implementing the FI program for linkage-to-care?
- How did the FI program for linkage-to-care change the environment at your site, if at all?
- How aware of the FI intervention for linkage-to-care were the members of your staff?

Impact of the FI Program on Clients

- In your opinion, what was the impact of the FI program for linkage-to-care on the clients at your site?
    - What did you think were the benefits of the program for your clients?
    - What did you think were the drawbacks of the program for your clients?
    - What were the differences among clients in how they reacted to the program?

Interactions with Clients during the FI Program

- How did you or your staff describe the FI program (coupons) for linkage-to-care to your clients?
    - What did you think your clients understood about why they were receiving the coupon?
    - Do you think your clients made the connection between linking to care, their health and the coupon? Why or why not?
- How did the FI program change the way you or your staff interacted with your clients?
- How did the FI program change the way you or your staff talked to your clients about linking to care?

End of the FI Program

- How do you think the end of the FI program for linkage-to-care has or will impact your site?
- How do you think the end of the FI program for linkage-to-care has or will impact your clients?
- If you were given the opportunity to offer FIs to patients for linkage-to-care in the future, would you do so? Why or why not?
- Is there anything about the FI program for linkage-to-care that you would change?

Site Migration

- Did you have clients who sought testing at your clinic specifically because they knew about the FI program for linkage-to-care?
    - If yes, can you give me some examples?

    - If yes, was this a problem for your site?
- How well do you think that people in the community knew about the FI program for linkage-to-care?

Do you have any additional thoughts about the FI program for linkage-to-care that you'd like to share with us?

SOC Test Site

(Note: these questions will only be asked to SOC Test/Care sites (FI Care or SOC Care that redeemed coupons)

IOR Opinions of the FI Program

- Although your site was randomized to the standard-of-care arm for the FI intervention for linkage-to-care, and thus didn't hand out any coupons, do you have any opinions about the intervention?
- If you were given the opportunity to offer FIs to your clients for linkage-to-care in the future, would you do so? Why or why not?

Site Migration

- How well do you think that people in the community knew about the FI program for linkage-to-care?

Do you have any additional thoughts about the FI program for linkage-to-care that you'd like to share with us?
